# Does the Construction of a Water Ecological Civilization City Improve Green Total Factor Productivity? Evidence from a Quasi-Natural Experiment in China

**DOI:** 10.3390/ijerph182211829

**Published:** 2021-11-11

**Authors:** Hongzhong Fan, Shuang Tao, Shujahat Haider Hashmi

**Affiliations:** 1 School of Economics, Huazhong University of Science and Technology, Wuhan 430074, China; hongzhong_fan@126.com; 2 Department of Management Sciences, Bahria University, Islamabad 75260, Pakistan; shujahat_hashmi@hotmail.com

**Keywords:** green total factor productivity, water ecological civilized city, environmental governance, compliance cost

## Abstract

Taking Water Ecological City Pilot (WECP) policy as a quasi-natural experiment, this paper adopts the PSM-DID method to investigate the impact of the WECP policy on the green total factor productivity (GTFP) of China’s prefecture-level cities. The results show that the implementation of the WECP policy significantly inhibits the improvement of GTFP. Furthermore, we find the implementation of the WECP policy has squeezed out government technological expenditures to some extent and aggravated the compliance cost of enterprises, which has not caused the “innovation compensation effect”, thus failing to improve GTFP. The heterogeneity analyses show that the policy effects vary with the imbalance of China’s regional development and resource endowments. Developed regions can better overcome the possible negative impact that comes with policy implementation. Governments need to formulate different policy strategies and plans from an overall macro perspective.

## 1. Introduction

Since the reform and opening up, China has achieved rapid economic growth relying on a model of high investment, high energy consumption, and high pollution. Undoubtedly, this rough development pattern has been accompanied by low efficiency in the use of natural resources and a continuous decline in environmental quality and environmental health [1]. In recent years, the Chinese government has committed to exploring the path of high-quality economic development to transform the original rough development model. Green development is an important guarantee for high-quality economic development. Essentially, green development is a sustainable development model with low energy consumption, low pollution, and high output. It emphasizes the people-oriented development concept, that is, while promoting economic growth, it must also achieve resource conservation and pollution reduction. Green total factor productivity (GTFP), as a correction to the traditional total factor productivity (traditional total factor productivity only considers the input constraints of capital, labor, and other production factors, and does not include the constraints of resource consumption and environmental pollution. In reality, resources and the environment are not only internal to economic development, but are is also a rigid constraint on the scale and speed of economic development [2]), is the main driving force and an important measurement index for realizing economic green transformation and development [3].

The effective use of resources, especially the use and protection of water resources, is a key factor that cannot be ignored in green development. China is a water shortage country whose per capita freshwater is only one-fourth of the world average. With the development of urbanization and industrialization in China, the water pollution caused by the rough development pattern has become a problem that cannot be ignored (the overall environmental quality of groundwater has improved after adopting a series of measures of pollution control. The evaluation shows that poor and extremely poor water account for 17.5% and 7.6% in 2019, an increase of 0.7% and decrease of 7.9% from 2016. However, the proportion of poor and extremely poor water for underground water is 66.9% and 18.8%, respectively, which means there is still a long way to go in the construction of water ecological civilization in China (the relative data come from “China Environmental Status Bulletin”, which is available at Ministry of Ecology and Environment of the People’s Republic of China (mee.gov.cn, accessed on 10 October 2020)). Water is a basic need of human life and production; deterioration of the water environment will not only damage the ecological environment and directly endanger people’s health, but also indirectly affect or even reduce production efficiency and hinder the economic development of society [4,5,6]. Nowadays, the green development model is being given great attention in China. Specifically, win-win strategies for the water ecological environment and economic development have been widely studied by scholars [7,8].

To explore the effective construction mode of water ecological civilization, the Chinese Ministry of Water Resources set up two batches of 105 water ecological civilization pilot cities with special policies in total from 2012 to 2014. The aim of the Water Ecological City Pilot (WECP) policy is to not only improve the urban environmental quality, but also to promote green productivity and achieve green development. However, is this WECP policy experiment effective? This question is very important for dealing with water pollution and promoting green development in developing countries.

To further answer the above questions, this paper adopts the PSM-DID method to study the real impact of the WECP policy, a kind of comprehensive water governance policy, on the GTFP among Chinese prefecture-level cities. It was found that WECP policy inhibited the growth of the city’s GTFP in the short run. The implementation of the WECP policy has squeezed out government technological expenditures to some extent and aggravated the compliance cost of enterprises, which has not caused the “innovation compensation effect”, thus failing to improve GTFP. Furthermore, the policy effects vary with the imbalance of China’s regional development and resource endowments. The WECP policy significantly inhibits the GTFP in small- and medium-sized cities. Compared with eastern and non-RB cities, the WECP policy exerts a more significant and negative impact on GTFP. These findings provide important guiding significance for government officials to rationally formulate and implement water governance policies.

The main contributions of this paper are as follows: first, to our knowledge, this is the first paper that takes the pilot policy of a water ecological civilization city as a quasi-natural experiment and examines the impact of water environmental governance on green total factor productivity using the PSM-DID method, which alleviates possible sample selection and endogenous problems. Second, this paper investigates the potential theoretical mechanism of the effect of WECP policy on GTFP from the perspective of fiscal expenditure and firm cost, and provides empirical evidence that supports the “compliance cost effect” rather than the “innovation compensation effect”. Third, this paper employs prefecture-level data and takes regional development level, city size, and resource endowment into consideration so that it partly fills the gap of the heterogeneity of policy effects that the past literature has rarely analyzed.

The structure of this paper is as follows: Section 2 reviews the relevant literature. Section 3 summarizes the background of the WECP policy and presents the theoretical mechanism between the policy and GTFP. Section 4 provides an introduction to the empirical strategy and data source of this study. Section 5 presents the empirical results and further discusses the theoretical mechanism and policy heterogeneity. Section 6 concludes this paper.

## 2. Literature Review

Two branches of literature relevant to our research exist. The first branch is the literature studying the relationship between environmental regulations and green total factor productivity, but have three inconsistent views. The first view is the “compliance cost effect”, which is based on neoclassical economics theory [9,10,11,12]. The implementation of environmental regulations will increase the pollution control costs of enterprises and even squeeze R&D investment funds, thereby inhibiting the improvement of corporate performance and GTFP. For example, Xie (2017) used China’s provincial panel data and found that the direct impact of environmental regulations on GTFP was negative in the short run, because non-environmental technology innovation may not promote GTFP due to its “crowding-effect” on production resources [13]. Moreover, employing the panel data of Chinese manufacturing industries during 2003–2014, Yuan and Xiang (2018) found that environmental regulation has crowded out R&D investment, and hindered GTFP in the short run [14]. Wu and You (2019) found that environmental regulations inhibited technological innovation at the national level, thus inhibiting green total factor productivity based on China’s provincial data from 2004 to 2015 [15].

The second view is the “innovation compensation effect”, which is based on the “Porter hypothesis” [16,17]. It believes that reasonable environmental regulations can stimulate enterprises to transform the traditional production pattern and carry out green innovation to offset the company’s environmental governance costs, thereby promoting the growth of GTFP. Many studies have supported this view. For example, Yuan and Xie (2015) employed the slack-based directional distance function and Luenberger productivity index to measure GTFP and its components of China’s industry, and the regression results indicated that environmental regulation could significantly improve the GTFP, which supports the “Porter Hypothesis” [18]. Chen et al. (2018) calculated the GTFP of China’s 36 industrial sectors and found that environmental regulation does improve the GTFP, while the driving effect of independent research and development on GTFP is obvious compared with technology importation [19].

The third type of view is that there is an uncertain or non-linear relationship between environmental regulations and GTFP. For example, Zhou et al. (2019) used the SBM-Luenberger productivity index to measure the GTFP of Chinese provinces and found that there is a nonlinear relationship between environmental regulation and green total factor productivity with the help of the panel threshold model [20]. Moreover, using panel data for manufacturing industry sectors from 2008 to 2015, Cao et al. (2020) found that environmental regulation exhibits a U-shaped nonlinear impact on GTFP and there is an initial inhibiting effect followed by a positive impact on green growth in the manufacturing industry as the intensity of environmental regulations increases [21].

However, nearly all of the above-mentioned studies constructed indicators to measure environmental regulations from some chosen aspects, such as total pollution fees, pollution control costs, industrial waste emissions, etc. The indicators constructed in this way inevitably lead to endogenous problems, such as reverse causation and missing variables, and ultimately lead to unreliable research conclusions. For example, according to Kuznets’ hypothesis, the rise of the national income may result in more attention being given to environmental pollution and lead to higher pollution fees at the initial time. It is difficult to identify the socio-economic effects of environmental regulations using the ordinary regression method with pollution fees as a proxy variable of environmental regulations.

The other branch of the relevant literature is mainly concerned with evaluating the economic effects of environmental policies or regulations. Fortunately, the difference in difference (DID) method treats an environmental policy shock as a quasi-natural experiment that may help researchers better identify the causal socio-economic effects of environmental regulations. More and more scholars are using the DID method to assess the implementation effects and economic effects of Chinese environmental policies. For example, Song et al. (2019) used the DID method to investigate the impact of low-carbon city construction on air quality and found that the policy has significantly reduced urban air pollution [22]. Cai et al. (2016) identified the causality between environmental regulation and foreign direct investment through the “Two Control Zones” implemented by the Chinese government in 1998 [23]. They found that tougher environmental regulation leads to less foreign direct investment. Taking China’s recent SO_2_ Emissions Trading Pilot as a quasi-natural experiment, Peng et al. (2021) identified the productivity effects of this market-based environmental regulation and suggested that the market-based environmental regulation has exerted significant productivity-enhancing effects across all types of industrial enterprises, with stronger effects associated with privately owned, more productive, and less pollution-intensive enterprises [24].

Some scholars have paid attention to the economic effects of Chinese water governance policies. Wang et al. (2018) focused on the prevention-and-control plans for the “three rivers and three lakes basins” designated by central government and found that although the water quality regulations forced many small heavily polluting firms to shut down, they had no statistically significant effects on surviving firms’ productivity because they were ineffective in reducing their COD emissions [7]. Chen et al. (2018) studied the consequences of spatially differentiated water pollution regulation related to the 11th (2006–2010) Five Year Plan in China and revealed that the regulation reduced pollution-intensive activity only in highly regulated areas [25]. Li et al. (2020) investigated the “River Chief” policy and implied that the implementation of this policy was not as effective as the government claimed [8].

Few researchers have studied the relationship between Chinese water governance policies and GTFP. The WECP policy is a comprehensive water governance policy shock aimed at promoting green development, and it provides a good quasi-natural experiment for us to use the DID method to study the relationship between Chinese water governance policies and GTFP. Compared with the existing literature, there is no doubt that this paper partly fills the research gaps related to water governance and has important practical significance for policymakers.

## 3. Institutional Background and Theoretical Analysis

### 3.1. Institutional Background

Historically, water resources have always played an important role in human life and development. As the largest developing country in the world, China has created a miracle of economic growth in the last decades. However, problems, such as water shortages, pollution, and deterioration of the water ecological environment, have become more and more serious. To deal with the urgent water problems, the Chinese government has started and made great efforts to construct water ecological civilization. The construction of water ecological civilization can effectively alleviate the pressure of water shortage and improve the efficiency of water use, thus promoting China’s green development [26].

**Figure 1:**
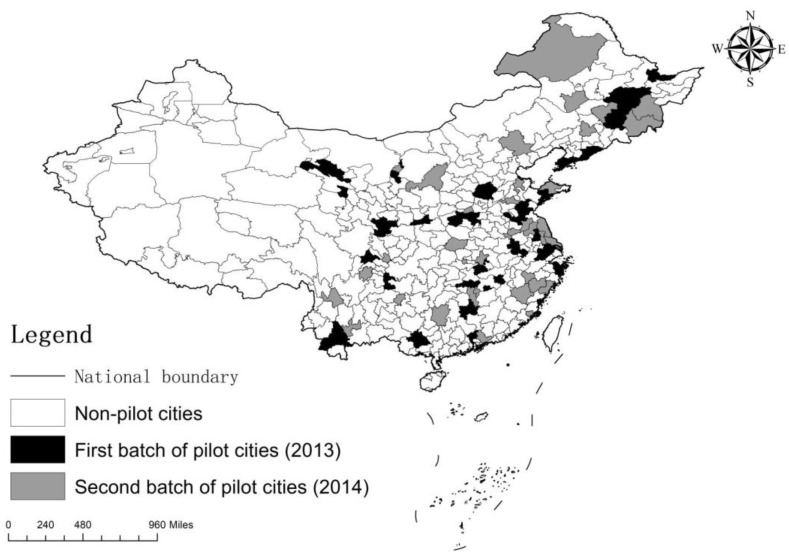
Spatial distributions of the two batches of water civilization construction pilot cities in China.

Under this context, to accelerate the construction of water ecological civilization, local governments have responded to the central government’s decision and deployment. In March 2013, the Ministry of Water Resources of the People’s Republic of China issued the document *“Notice of the Ministry of Water Resources on Launching the Pilot Work for the Construction of National Water Ecological Civilization”*, and launched the pilot work for the construction of water ecological civilization nationwide. In August of the same year, the Ministry of Water Resources announced the first batch of 45 pilot cities for the construction of aquatic ecological civilization. In June 2014, the Ministry of Water Resources established the second batch of 59 pilot cities for water ecological civilization construction. By the end of May 2019, most of the pilot cities had successfully passed the inspection and acceptance. The specific pilot cities are shown in Table 1. Figure 1 demonstrates the distribution of the pilot cities.

The core content of the WECP policy is to establish a strict water resources management system that delineates policy objectives from the perspectives of water resources protection, water pollution prevention, and water ecological restoration, etc. For example, the pilot cities clearly included the “three red lines” and “four systems” (in 2012, the State Council issued the “Opinions on Implementing the Strictest Water Resources Management System” and proposed the “three red lines” and “four systems” of water resources management to improve the water environment and achieve sustainable development. The “three red lines” are the red lines for the development and utilization of water resources, the red lines for water efficiency control, and the red lines for pollution control in water function zones. “Four systems” is the corresponding management system. Detailed information is available at The State Council of the People’s Republic of China (www.gov.cn, accessed on 15 October 2020)).

As the government assessment basis is the key points of the government’s water resources management system, the city production activities are evaluated in terms of economic efficiency and ecological effects. At the same time, the water pilot policy has established a series of systems, such as a water rights system, a water price system, and an ecological compensation mechanism, to optimize the allocation of water resources and improve water use efficiency in the urban production process. Essentially, the WECP policy is a comprehensive water governance policy with the goal of ecological improvement and sustainable economic development.

### 3.2. Theoretical Analysis

At present, there is no consistent conclusion on the relationship between environmental policies and GTFP. Although the WECP policy aims to improve the water ecological environment and achieve green development, has this policy effectively promoted the GTFP? With this question, we analyzed the potential impact mechanism between WECP policy and GTFP based on the relevant policy background and content.

We first propose the “Crowding-out effect”. According to the water pilot policy preparation documents, each pilot city must also do a good job of water system connectivity, water conservation, and other preliminary engineering measures based on water pollution control, which mainly depend on government expenditure. So far, the cumulative investment in WECP construction has reached 750 billion yuan according to related reports. In the case of limited government revenue, the implementation of the WECP policy may inevitably squeeze the government’s productive expenditures on related technological innovation infrastructure. For example, it may reduce government support for venture capital, universities, scientific research institutes, and common technology research projects, etc. Additionally, incentives for technological innovation of enterprises may be hurt due to a lack of government financial support. Considering these circumstances, the implementation of the WECP policy may weaken the pilot cities’ innovation capability in the short term, finally resulting in a decline in GTFP in the short term.

The second is the “Cost effect”, which is based on the traditional review supported by Jaffe et al. (1995) [9] and Greenstone (2012) [11], who think that environmental regulations require polluting facilities to undertake abatement activities, and thus, they impose extra costs on enterprises and further hinder productivity. As a strict and comprehensive water resource regulation policy, the WECP policy also requires cities to formulate main tasks and establish related water ecological compensation mechanisms and water pollution control mechanisms. For example, the pilot cities have formulated strict sewage discharge and water utilization efficiency indicators, and strictly controlled the water use activities of enterprises. On the one hand, these measures will lead to some serious pollution enterprises passively reducing production or even shutting down to temporarily meet the strict requirements of environmental protection regulations [27]. On the other hand, these enterprises will not only increase pollution control expenditures, but also need to increase R&D investment related to energy conservation and emission reduction. These actions responding to water regulations undoubtedly increase the compliance cost of the enterprises and decrease their GTFP [28], which is ultimately reflected in the reduction of GTFP in the pilot cities.

Based on the above analysis, we, therefore, proposed the following hypotheses:

**Hypothesis** **1** **(H1).**  *The construction of a water ecological civilization city reduces the GTFP*.

**Hypothesis** **2** **(H2).**  *The construction of a water ecological civilization city negatively affects the GTFP through two kinds of mechanisms: the “crowding-out effect” and “cost effect”.*

## 4. Methodology and Data

### 4.1. Calculation of the GTFP

Green total factor productivity covers labor, capital, and energy input. At the same time, it takes into account the increase in expected output and the decrease in undesired output. The measurement of this indicator is related to the energy and environmental problems that China needs to solve in future economic development. Referring to Färe et al. (2007) [29], this paper constructs a set of production possibilities including expected output and undesired output and uses the Malmquist–Luenberger index based on the non-radial SBM directional distance model to calculate the green total factor productivity of 183 cities in China from 2006 to 2018. The basic calculation method is as follows:

Regard each city as a decision-making unit, where each decision-making unit includes input, “good” output, and “bad” output. Suppose that each city uses M kinds of inputs $x=\left(x_{1},\ldots ,x_{m},\ldots ,x_{M}\right)\in R_{M}^{+}$ to produce N kinds of “good” outputs $y=\left(y_{1},\ldots ,y_{n},\ldots ,y_{N}\right)\in R_{N}^{+}$ and discharge J types of “bad” output $b=\left(b_{1},\ldots ,b_{j},\ldots ,b_{J}\right)\in R_{J}^{+}$. The production possibility set reflecting the environmental technology is: $p^{t}\left(x\right)=\left\{\left(x_{t},y_{t},b_{t}\right):x_{t}\right\}$, and there are some basic assumptions of the production possibility set that need to be satisfied: the set is a closed set and a bounded set, the input and the expected output are freely disposable, Null-jointness Axiom, and weak disposability of the output. Therefore, using data envelopment analysis (DEA), the environmental technology model can be expressed as:

(1) $$P^{t}(x^{t})=\left\{\left(y^{t},b^{t}\right):\overset{I}{\underset{i=1}{\sum }}z_{i}^{t}y_{in}^{t}\geq y_{in}^{t},\forall n;\overset{I}{\underset{i=1}{\sum }}z_{i}^{t}b_{ij}^{t}=b_{ij}^{t},\forall_{j};\overset{I}{\underset{i=1}{\sum }}z_{i}^{t}x_{im}^{t}\leq x_{im}^{t},\forall m;\overset{I}{\underset{i=1}{\sum }}z_{i}^{t}=1,z_{i}^{t}\geq 0,\forall i\right\}$$

Among them, i=1,2,3,…,I represents the corresponding cities; t=1,2,…,T represents the period; and $z_{i}^{t}$ represents the weight of each cross-sectional observation value. Referring to the method of Tone (2004) [30] and Fukuyama and Weber (2009) [31], this study defines the SBM direction distance function including environmental variables as:

(2) $$\vec{S_{V}^{t}}(x^{t,i^{\prime }},y^{t,i^{\prime }},b^{t,i^{\prime }},g^{x},g^{y},g^{b})=\max_{s_{x},s_{y},s_{b}}\frac{\frac{1}{M}\sum_{m=1}^{M}\frac{s_{m}^{x}}{g_{m}^{x}}+\frac{1}{N+J}\left(\sum_{n=1}^{N}\frac{s_{n}^{y}}{g_{n}^{y}}+\sum_{j=1}^{J}\frac{s_{j}^{b}}{g_{g_{j}}^{b}}\right)}{2}$$

Restrictions:

$$\overset{I}{\underset{i=1}{\sum }}z_{i}^{t}x_{im}^{t}+s_{m}^{x}=x_{i^{\prime }m}^{t},\forall m$$

$$\overset{I}{\underset{i=1}{\sum }}z_{i}^{t}x_{in}^{t}-s_{n}^{x}=x_{i^{\prime }n}^{t}$$

$$\overset{I}{\underset{i=1}{\sum }}z_{i}^{t}b_{ij}^{t}+s_{j}^{b}=b_{i^{\prime }j}^{t}$$

$$\overset{I}{\underset{i=1}{\sum }}z_{i}^{t}=1,z_{i}^{t}\geq 0,\forall i$$

$$s_{m}^{x}\geq 0,\forall m;s_{n}^{y}\geq 0,\forall n;s_{j}^{b}\geq 0,\forall j$$

The input and output vectors of city i are $\left({x^{t,i}}^{\prime },{y^{t,i}}^{\prime },{b^{t,i}}^{\prime }\right)$, the expected output expansion and undesired output. The positive direction vector of the sum input compression is $\left(g^{x},g^{y},g^{b}\right)$, and the relaxation vector of the input and output is $\left(s_{m}^{x},s_{n}^{y},s_{j}^{b}\right)$.

According to the method proposed by Chung et al. (1997) [32], this paper expresses the green total factor productivity index ML from period t to period t+1 as:

(3) $$ML_{t}^{t+1}={\left\{\frac{1+\vec{S_{0}^{t}}\left(x^{t},y^{t},b^{t};g^{t}\right)}{1+\vec{S_{0}^{t}}\left(x^{t+1},y^{t+1},b^{t+1};g^{t+1}\right)}\times \frac{1+\vec{S_{0}^{t+1}}\left(x^{t},y^{t},b^{t};g^{t}\right)}{1+\vec{S_{0}^{t+1}}\left(x^{t+1},y^{t+1},b^{t+1};g^{t+1}\right)}\right\}}^{\frac{1}{2}}\;$$

Among them, $\vec{S_{0}^{t}}\left(x^{t},y^{t},b^{t};g^{t}\right)$,$\;\vec{S_{0}^{t+1}}\left(x^{t+1},y^{t+1},b^{t+1};g^{t+1}\right)$ represent the current directional distance function of period t and t+1, respectively; and $\vec{S_{0}^{t}}\left(x^{t+1},y^{t+1},b^{t+1};g^{t+1}\right)$ and $\vec{S_{0}^{t+1}}\left(x^{t},y^{t},b^{t};g^{t}\right)$ is the mixed directional distance function. The former represents the directional distance function of t+1 production activities based on the production frontier of the t period, and the latter represents the directional distance of the t period based on the t+1 production frontier. When $ML_{t}^{t+1}>1$, it means that from period t to period t+1, the GTFP of this area has been improved; otherwise, the GTFP index of this area has not been improved.

### 4.2. Empirical Model

The issue studied in this paper is whether the WECP policy can help improve the urban GTFP. To solve the endogenous problems commonly faced in the literature, this paper applies this pilot policy as a “quasi-natural experiment” to construct a double difference: the first level of the difference comes from the city level, and the second level of the difference comes from the year level. 

However, due to the heterogeneity of different cities, it is not easy for different cities to meet the condition of consistent time effects. Moreover, the selection of water pilot cities may not be completely random, because it is possibly affected by the city’s development status and water pollution, which results in a selection bias. This bias may cause the explanatory variables to be correlated with the residuals, leading to an endogenous problem [33]. Under this condition, simply performing DID regression will produce biased estimates. Therefore, this paper first employs the propensity score matching method (PSM) developed by Heckman (1976) [34] and Rosenbam and Rubin (1983) [35], which can make “similarity” between the treatment group and the control group, to match the samples, and then uses the matched samples to mitigate the sample selection bias. It is better to use the PSM-DID method to research such policy effects [36]. So, this paper adopts the PSM-DID method to assess the net effects of the WECP policy on GTFP.

Specifically, two batches of the pilot water ecology policy have been implemented and the pilot policies were carried out in 2013 and 2014, respectively (Jinan was the only pilot city in 2012 and was excluded in the subsequent analysis). The assessment of this paper is mainly based on the sample of pilot cities in 2013. So, the water ecology pilot cities established in 2013 are defined as the treatment group, and the non-pilot cities are defined as the control group. Furthermore, this paper uses pilot cities in 2014 to perform the robustness test. Due to the lag in policy implementation and the short period of the data sample in the experimental period, the effects of the water pilot cities studied in this paper are all short-term impacts, and long-term impacts require further improved data to be identified.

The regression model based on the PSM-DID method is set as follows:

(4) $$GTFP_{it}=\alpha_{0}+\alpha_{1}Treated\times Time+\sum_{j=1}^{n}\beta_{j}\times Control_{jit}+\mu_{i}+\lambda_{t}+\varepsilon_{it}$$

Among them, $GTFP_{it}$ is the ML productivity index calculated above, which measures the green total factor productivity; the core explanatory variable is Treated×Time, in which Treated represents a dummy variable that takes on a value 1 if the city is one of the WECP cities in 2013, and 0 otherwise; and Time represents the time dummy variable that takes on a value of 1 if the policy is implemented in 2012 or later, and 0 otherwise. The coefficient $\alpha_{1}$ captures the net policy effect. $Control_{jit}$ represents a series of control variables at the city level and $\mu_{i}$ represents the individual fixed effect, which controls underserved factors that do not vary with time, such as geographic location, etc. $\lambda_{t}$ represents the time fixed effect, which controls underserved factors that do not vary with individual characteristics, such as macroeconomics changes in the situation, etc.$\;\varepsilon_{it}$ is a random disturbance term.

### 4.3. Variables and Data Resource

#### 4.3.1. Indicators for GTFP Calculation

The indicators for calculating GTFP include input, expected output, and undesired output indicators. Input indicators include labor input, which is represented by the total number of employees in each city over the year; capital input, which is measured by capital stock; and water input, which is measured by annual industrial water consumption. We use the most widely used perpetual inventory method to estimate the capital stock, and the specific method is as follows. According to the processing method of Ke (2012) [37], he defines $K_{t}=K_{t-1}\times \left(1-\delta \right)+\frac{I_{t}+I_{t-1}+I_{t-2}}{3}$, where $K_{t}$ represents the capital investment in year t, and $I_{t}$ is the fixed asset investment amount at a constant price in year t. Following Shan (2008) [38], we set the depreciation rate δ to 10.96%. The investment amount in the base period is $K_{0}=I_{0}\times \left(1+g\right)/\left(g+\delta \right)$ and is determined, where g is the average growth rate of constant investment $I_{0}$, and $I_{0}$ is the constant price fixed asset investment in the initial year. For the energy input, we draw on the practice of Qin (2014) and use the city’s annual electricity consumption to express it [39].

Output indicators: Expected output is represented by urban gross national product; we use urban actual GDP calculated at constant prices in 2005 to measure the expected output of each city. Undesired output is represented by a comprehensive index combined with wastewater discharge, sulfur dioxide discharge, and soot discharge. Here, we use the entropy method to determine the weight of undesired output to calculate the comprehensive index of undesired output.

#### 4.3.2. Control Variables

Concerning relevant theories and literature, we select the following control variables: The economic development level (*Lnrgdp*) is represented in terms of real GDP per capita and taken as a logarithm. The level of urban human capital (*Human*) is measured by the proportion of the number of students in ordinary colleges and universities in the total population. As the development of the financial market has a non-negligible effect on the environment and productivity, this paper uses the proportion of financial institution loan balance to GDP at the end of the year to measure the level of financial development (*Finance*). The infrastructure (*Infrastr*) is measured by the per capita area of the urban road area [40]. The industrial structure (*Industr*) is represented as the ratio of the secondary industry’s added value to the tertiary industry’s added value.

The data in this study mainly come from the official statistics of the Ministry of Water Resources’ policy documents on the development of water ecological civilized cities, the *“China City Statistical Yearbook”* and *“China Statistical Yearbook”*. In the process of selecting the treatment group and the control group, we performed the following treatments: (1) in the implementation of the water pilot policy, some pilot areas are not the prefecture-level city, but a certain county or district within the prefecture-level city (such as Changting County, Longyan City). If such prefecture-level cities are included in the analysis, the estimation results will be inaccurate. Therefore, this type of prefecture-level city is excluded from the sample. (2) Considering the special administrative divisions, four municipalities directly under the central government are eliminated in this paper [41]. (3) To extend the range of policy estimation as much as possible, we use the water pilot cities in 2013 as the treatment group as the benchmark regression. At the same time, to ensure that the estimated result is the net effect of the 2013 pilot policy, we exclude the newly emerged pilot cities in 2014. (4) We removed some prefecture-level cities with serious data missing, and supplement cities with only a few missing data through linear fitting and data smoothing. At the same time, all data involving prices are deflated to the real constant price in 2005 based on the GDP deflator. Finally, a balanced panel data composed of 183 cities in China from 2006 to 2018 is obtained. The descriptive statistics of specific variables are shown in Table 2.

## 5. Empirical Results and Discussion

### 5.1. Sample Matching

When using the PSM-DID method, the first step is to find a control group for the treatment group through the PSM method. The specific processes are as follows: (1) estimating the propensity score of the treatment group and control group cities through the logit model, respectively; (2) following the research of Abadie et al. (2004) [42], we choose the one-to-one nearest neighbor matching method to match the sample cities according to the propensity score value. Moreover, we selected the economic development level, urban human capital, infrastructure level, industrial structure, and financial development level as the matching characteristic variables.

**Figure 2:**
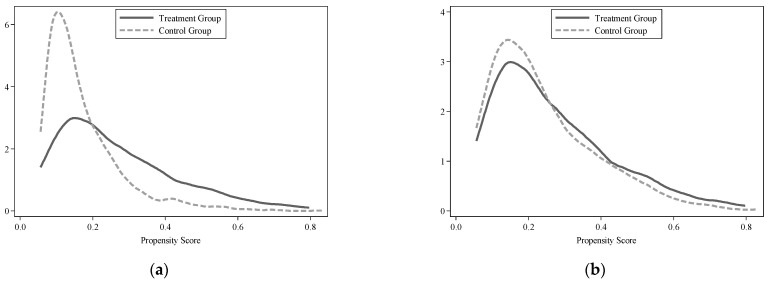
Distribution of propensity scores by treatment and control groups: before and after the neatest-neighbor PSM. (**a**) Before matching, (**b**) after matching.

To ensure the reliability of the matching results, the common support test and the balancing test are performed before our regression. Table 3 reports the results of the sample data balancing test after the matching. It can be seen that after the matching, there is no significant system difference between the treatment group and control group, and the absolute value of the standardized deviation of all variables after matching is less than 15%, indicating that the sample distributions of the treatment group and the control group have good consistency and satisfy the balance assumption of PSM. Besides, comparing the kernel density distributions of propensity scores before and after matching (see Figure 2), we can also find that there is a big difference in the distribution of propensity scores between the pre-matching treatment group and the control group. However, after the matching, the difference between the two is significantly reduced and the trend is the same, which once again proves the balance of the data after the match.

### 5.2. Basic Empirical Results

This paper estimated the overall effect of the pilot policy of water ecological civilization city on GTFP by a full sample estimation. Before carrying out PSM-DID estimation, we first conducted the Hausman test. According to the test results, we chose a two-way fixed-effects model for regression. The PSM-DID estimation results are shown in Table 4. The (1) to (4) columns in Table 4 are the results of gradually adding control variables. Among them, the coefficients of the interaction term Treated×Time reflect the impact of the WECP policy on the GTFP. Whether control variables are added, the coefficients of the Treated×Time term are significantly negative at the 1% confidence level, and the coefficient is stable between −0.0791 and −0.0956. This means that the WECP policy has a significant inhibitory effect on GTFP. As for the regression results of control variables, the coefficients of *Lnrgdp* and *Finance* show a significant positive effect on GTFP at a 1% confidence level, which means that the city’s economic development level and financial development play an important role in promoting GTFP. The more developed the city, the more willing it is to achieve green development. The coefficients of other control variables are almost positive but insignificant, which means that the infrastructure, technological innovation, and industrial structure upgrading have no significant impact on GTFP. It may be due to the time lag between these construction activities and GTFP.

### 5.3. Placebo Test

To ensure the robustness of the above regression results, this paper conducts placebo tests from the following three aspects.

#### 5.3.1. Advancing the Policy Implementation Time

To verify that it is the implementation of WECP policy that affects the GTFP and eliminate coincidences, this paper constructs a counterfactual test [43], which will advance the establishment of water pilot cities by one year ($Treated\times time_{1}$), two years ($Treated\times time_{2}$), and three years ($Treated\times time_{3}$). If the regression coefficient is not significant, it can be proved that it is the WECP policy that indeed has a significant negative impact on GTFP. Table 5 presents the counterfactual test results of the policy. The first column is the benchmark regression result, and columns (2), (3), and (4) are the results of one year, two years, and three years in advance. It can be concluded that after the implementation time of the WECP policy is advanced, all the interaction coefficients are insignificant and there is no systematic error that proves that the benchmark regression results are credible.

#### 5.3.2. Changing the Sample Period

The sample period selected in this paper is 2006–2018, and the impact of different policy shocks during the sample period (such as the pollution charges reform on $SO_{2}$ in 2007, the low-carbon city construction policy in 2010, etc.) may affect the estimation results of this paper. To eliminate the impact of potential policy effects, we conduct the robustness test, changing the time interval of the regression [36]. The specific approach is to take the policy implementation time 2013 as the middle point, and select samples of 1, 2, and 3 years before and after the regression. If the significance of regression coefficients hardly changes, the research results are relatively stable. From the regression results in Table 6, though the magnitude of the coefficient changed a little bit, we can see that the estimated coefficients under different sample periods are significantly negative and relatively stable. So, it can be concluded that the results support the previous conclusions and prove that our baseline estimation results are robust.

#### 5.3.3. Changing the Treatment Group Sample

Since there are mainly two batches of water ecological civilization cities in 2013 and 2014, this paper uses the 2014 pilot cities as a robustness test. After excluding the pilot cities in 2013, the regression results of the PSM-DID estimation are shown in Table 7. The significance of the interaction coefficients has hardly changed, indicating that the pilot construction of water ecological civilization cities significantly reduced the green total factor productivity, which further proved the robustness of the conclusion of this paper.

### 5.4. Influencing Mechanism Analysis

The above benchmark regression and robustness tests both show that the WECP policy will decrease GTFP. In this section, this paper further examines the possible theoretical mechanism behind this negative relationship. As analyzed in Section 3.2, the WECP policy negatively affects GTFP through two kinds of channels: the “crowding-out effect” and “cost effect”. To further verify the existence of these effects, we adopt a two-stage mediating effect model for verification [44].

The first stage is to verify the driving effect of WECP policy on the two major effects. A mediation model is constructed to test the effects of policy variables on mediating variables, see model (5). If $\alpha_{1}$ is not significant, the test of the mediating effect will be stopped; otherwise, it means that the policy variable has a significant effect on the mediating variables, and the second stage will be entered:

(5) $$Govetec_{it}\left(Comcost_{it}\right)=\alpha_{0}+\alpha_{1}Treated\times Time+\overset{n}{\underset{j=1}{\sum }}\theta_{j}\times Control_{jit}+\mu_{i}+\lambda_{t}+\varepsilon_{it}$$

The second stage is to verify the effect of the two major effects of the WECP policy on the GTFP by setting up a comprehensive model (6) based on the mediating model (5). If $\beta_{2}$ is insignificant, there is no mediating effect. Otherwise, there is a mediating effect, regardless of whether $\beta_{1}$ is significant. If $\beta_{1}$ is insignificant, it indicates that the mediating variable is the only transmission path of the policy variable to GTFP. Otherwise, other transmission paths exist:

(6) $$GTFP_{it}=\beta_{0}+\beta_{1}Treated\times Time+\beta_{2}Govetec_{it}\left(Comcost_{it}\right)+\overset{n}{\underset{j=1}{\sum }}\theta_{j}\times Control_{jit}+\mu_{i}+\lambda_{t}+\varepsilon_{it}\;$$

In model (5), $Govetec_{it},\;Comcost_{it}$ represent the two mediating variables that take the logarithmic form. $Govetec_{it}$ represents government spending on science and technology. The data of $Govetec_{it}$ comes from *China City Statistical Yearbooks* from 2006 to 2018. $Comcost_{it}$ represents the compliance cost brought by the urban environmental governance. Intuitively, the changes in cost and productivity brought about by urban environmental policies are mainly reflected in the activities of enterprises. So, this paper adopts the environmental protection expenditure of enterprises aggregated to the city level to measure the compliance cost. The data are collected from annual financial reports of listed companies in China.

The results of the above mechanism test are shown in Table 8. We first test the “crowding-out effect”. Columns (1) and (2) show that the WECP policy can significantly decrease government technical investment whether control variables are added or not. Columns (3) and (4) test the effect of government technological expenditures on GTFP. The coefficient of Treated×Time is significantly negative while the coefficient of $Govetec_{it}$ is significantly positive, suggesting that government technical expenditures can significantly improve GTFP. Combining the four columns of the results, it can be concluded that the implementation of the WECP policy squeezes out the scientific and technological expenditures of governments and weakens technological innovation, finally inhibiting the GTFP in pilot cities. 

The remaining four columns test the “compliance cost effect” between the WECP policy and GTFP. Columns (5) and (6) examine the impact of the WECP policy on compliance cost. The positive significant influencing coefficients of Treated×Time mean that the WECP policy raises the compliance cost in the pilot cities. Columns (7) and (8) test the effect of compliance cost on GTFP. The coefficients of $Comcost_{it}$ are significantly negative. Hence, combining the results from columns (5) to (8), we can conclude that the WECP policy has increased the compliance costs of enterprises, thereby reducing GTFP. 

To summarize, hypothesis 2 is supported by the results of the mediating models. Although the expected goal of the WECP policy is to achieve green transformation and sustainable development, we provide empirical evidence that the pilot policy has decreased the technological innovation capabilities and increased the compliance cost of pilot cities, ultimately failing to achieve the goal of green development. In other words, there is considerable room for the WECP policy to be further perfected and strengthened. 

### 5.5. Heterogeneity Analysis

Due to the different development scales and resource endowments among the cities that may affect the policy effect, this paper further conducts the heterogeneity analyses from three aspects: city size, geographic location, and resource endowments.

#### 5.5.1. City Size Heterogeneity

Cities of different sizes have different levels of development, and large cities have significant agglomeration effects compared to small cities [45]. The advantages of labor pools, intermediate input sharing, and knowledge and technology spillovers formed by such agglomeration effects [46] not only help improve resource allocation but also optimize the adjustment of the industrial structure and improve the production efficiency of the city; however, the rapid development brought about by the agglomeration economy is also accompanied by environmental and resource consumption issues that cannot be ignored. Therefore, it is necessary to study the heterogeneous impact of the WECP policy from the perspective of city size.

This paper refers to the city size classification issued in the *Notice on Adjustment of City Size Classification Standard* in 2014 and divides China’s cities into five categories (the detailed information is available at http://www.gov.cn/zhengce/content/2014-11/20/content_9225.htm, accessed on 10 November 2020). Among them, cities with a permanent population exceeding 10 million municipal districts are classified as mega cities, cities with a population between 5 and 10 million are super cities, cities with a population between 1 and 5 million are big cities, cities with a population between 0.5 and 1 million are medium cities, and all others are small cities. Given the small number of mega cities, this paper further reclassified cities into four groups. 

The specific results are shown in Table A1 in Appendix A. It can be seen that for small- and medium-sized cities, the influencing coefficients of Treated×Time are significantly negative, while for big and super cities insignificant. Therefore, it can be included that the WECP policy has a greater inhibitory effect on GTFP in smaller cities. This may be due to the fact that the innovation and productivity advantages brought about by the agglomeration effect in big cities will compensate to a certain extent for the rising costs when facing the WECP policy.

#### 5.5.2. Regional Heterogeneity

In this subsection, we test the heterogeneity impact of the WECP policy considering geographic location and resource endowments.

First, cities in different geographical locations have different economic development conditions, and their industry distribution is also significantly different. The cities in the eastern region have relatively high economic development levels, are dominated by capital-intensive and technology-intensive industries, and have relatively high developed service industries while the cities in the western region mainly contain labor-intensive industries. Therefore, the effect of WECP policy on GTFP may vary across cities in different locations. 

Second, considering the different natural resource endowments of each city, the industrial distribution and the competition mechanism among enterprises in these cities show huge differences. For example, some heavily polluting enterprises that rely on fossil energy will be more likely to be located in a city with rich natural resources, such as coal. Therefore, when facing environmental regulations, cities with different resource endowments will be affected to varying degrees. According to the *“Notice of the State Council on Issuing the National Sustainable Development Plan for Resource-Based Cities (2013–2020)”* (the detailed information is available at http://www.gov.cn/zwgk/2013-12/03/content_2540070.htm, accessed on 12 October 2021), we divided the sample cities into two groups, which are listed as resource-based (RB) and non-RB. The more specific classification of RB cities includes four types: growth, mature, decline, and regenerative cities.

The results of the regional heterogeneity tests are shown in Table A2 in Appendix A. Columns (1) to (3) show that the impact coefficients of the implementation of the WECP policy on the GTFP are significantly negative at a significance level of 5% and 10% in the western and central regions, respectively, while the impact of the WECP policy in the eastern regions is insignificant. The results indicate that it is easier for eastern cities to implement the construction of water ecological civilization and achieve the expected goals with their competitive and economic advantages. To a certain extent, they can easily shackle the constraints of environmental regulations brought by WECP policy, thereby offsetting its inhibitory effect on GTFP. The western and central cities are still facing greater pressure on the cost of water environment governance and investment pressure on the construction of water ecological cities, which inhibits the improvement of GTFP in the short run. Besides, columns (4) and (5) show that the WECP policy has a more significant impact on RB cities compared with non-RB cities. The coefficient of the Treated×Time term is significantly negative at the 5% confidence level in RB cities. It is not difficult to understand that polluting industries are more likely to be located in RB cities. Therefore, when facing WECP policy, enterprises of such industries are more likely to face rising governance costs and more vulnerable to the impact of WECP policy, which ultimately leads to the decrease in GTFP.

## 6. Conclusions and Recommendations

In the context of China’s economic green transformation and development, this paper takes the water ecological civilization city pilot policy as a quasi-natural experiment and uses the PSM-DID method to investigate the impact of WECP policy on GTFP and draws the following conclusions. 

First, the baseline model and robustness tests show that the WECP policy has significantly inhibited the growth of the city’s GTFP in the short run. Second, this paper finds no evidence supporting the Porter Hypothesis because of the “crowding-out effect” and “cost effect”, which are the two main transmission mechanisms for WECP policy to negatively affect GTFP. On the one hand, the implementation of the WECP policy has led the local government to increase a lot of infrastructure expenditure related to the water environment and squeeze out quite technological expenditures, which suppress the city’s innovation driving force, thereby failing to improve GTFP. On the other hand, the environmental constraints brought about by the WECP policy significantly increase the compliance costs of the enterprises, and thus reduce the GTFP in pilot cities. Third, the impact of the WECP policy on GTFP shows great heterogeneity in city size and location, and resource endowments. The WECP policy significantly inhibits the GTFP in small- and medium-sized cities. Compared with eastern and non-RB cities, the WECP policy exerts a more significant and negative impact on GTFP. These findings indicate that developed regions are more able to withstand the short-term pains brought about by the policy implementation and are easier to achieve expected goals. 

It is of great significance to think about how to overcome the possible negative impact that comes with the policy implementation. We then proposed the following policy recommendations based on our research results. First, the empirical results in this paper indicate that the implementation of WECP policies hinders the improvement of innovation capabilities and increases the burden on enterprises. So, it is necessary for local government to rationally allocate financial resources and provide corresponding financial subsidies to relevant enterprises, which can accelerate the improvement of the city’s innovation capabilities. Second, the heterogeneity analyses in this paper show that the WECP policy mainly affects the cities in central and western regions, and small and RB cities are more likely to be negatively affected. Therefore, governments need to formulate different policy strategies and plans for different locations and endowments from an overall macro perspective. For example, the government should implement differentiated policy intensities for resource-based and non-resource-based cities. Only appropriate policy strategies that conform to the actual conditions of the city can effectively overcome the possible negative policy effects and realize the expected benefits.

Although this paper has discovered and discussed the negative relationship between WECP policy and GTFP in the short run, further research is necessary. Due to the limitation of the data, this paper only considers the short-term impact of the WECP policy, and further study can be designed to explore the dynamic effects in the long run with more complete data later. 

## Figures and Tables

**Table 1:** Lists of water ecological civilization pilot cities.

Time	Pilot Cities
August 2013 (46 cities)	Miyun County, Wuqing District, Handan, Xingtai, Wuhai, Dalian, Dandong, Jilin, Hegang, Harbin, Qingpu District, Xuzhou, Yangzhou, Suzhou, Wuxi, Ningbo, Huzhou, Wuhu, Hefei, Changting County, Nanchang, Xinyu, Qingdao, Linyi, Zhengzhou, Luoyang, Xuchang, Xianning, Ezhou, Changsha, Chenzhou, Guangzhou, Dongguan, Nanning, Qionghai, Yongchuan District, Chengdu, Luzhou, Qianxinan Autonomous Prefecture, Pu’er, Xi’an, Zhangye, Longnan, Xining, Yinchuan
June 2014 (59 cities)	Mentougou District, Yanqing County, Jixian County, Chengde, Hulunbuir, Tieling, Yanbian, Changchun, Baicheng, Mudanjiang, Minhang District, Nantong, Huai’an, Taizhou, Suqian, Yancheng, Wenzhou, Quzhou, Jiaxing, Lishui, Bengbu, Huainan, Quanjiao County, Lixin County, Putian, Nanping, Pingxiang, Binzhou, Tai’an, Yantai, Jiaozuo, Nanyang, Xiangyang, Qianjiang, Wuhan, Fenghuang County, Zhejiang Dong Autonomous County, Zhuzhou, Huizhou, Zhuhai, Yulin, Guilin, Baoting Li and Miao Autonomous County, Bishan County, Liangping County, Suining, Leshan, Guiyang, Qiannan Autonomous Prefecture, Yuxi, Lijiang, Yangling Demonstration Area, Dunhuang, Haibei Autonomous Prefecture, Shizuishan, Loufan County, Tekesi County, Wujiaqu County, Naqu Region

Note: The data are derived from the website of the Water Resources Ministry (http://www.mwr.gov.cn/, accessed on 15 October 2020). Jinan was the only pilot city that was set in October 2012.

**Table 2:** Descriptive statistics.

	Full Sample			Treatment Group			Control Group		
Variable	Obs.	Mean	Std.Dev.	Obs.	Mean	Std.Dev.	Obs.	Mean	Std.Dev.
*GTFP*	2353	1.074	0.344	442	1.057	0.179	1911	1.078	0.372
*Finance*	2353	1.478	1.016	442	2.034	1.404	1911	1.35	0.853
*Human*	2353	0.0501	0.043	442	0.0695	0.0514	1911	0.0457	0.0395
*Infrastr*	2353	12.46	7.123	442	14.89	9.485	1911	11.9	6.326
*Industr*	2353	1.35	0.907	442	1.151	0.525	1911	1.396	0.969
*Lnrgdp*	2353	10.74	0.656	442	10.99	0.594	1911	10.69	0.656

**Table 3:** Balancing test results.

	Unmatched	Mean		%Bias	%Bias	*t*-Test	
Variable	Matched	Treated	Control		Reduction	*t*-Value	*p*-Value
*Lnrgdp*	U	10.990	10.686	48.6		8.94	0.000
	M	10.988	10.927	9.6	80.2	1.46	0.146
*Human*	U	0.070	0.046	52.1		10.78	0.000
	M	0.069	0.069	−1.1	97.8	−0.15	0.879
*Finance*	U	2.034	1.350	58.9	95.2	13.22	0.000
	M	2.012	2.045	−2.8		−0.34	0.732
*Industr*	U	1.151	1.396	−31.4		−5.13	0.000
	M	1.154	1.131	2.9	90.6	0.49	0.627
*Infrastr*	U	14.894	11.901	37.1		8.07	0.000
	M	14.890	15.000	−1.4	96.3	−0.19	0.852

**Table 4:** Basic regression results using PSM-DID.

	(1)	(2)	(3)	(4)
*Treated* × *Time*	−0.0791 ***	−0.0827 ***	−0.0956 ***	−0.0935 ***
	(0.030)	(0.030)	(0.030)	(0.030)
*Lnrgdp*		0.5257 ***	0.5726 ***	0.5628 ***
		(0.131)	(0.133)	(0.133)
*Infrastr*		−0.0014	0.0011	−0.0019
		(0.002)	(0.002)	(0.002)
*Industr*			−0.0070	−0.0073
			(0.013)	(0.013)
*Finance*			0.0381 ***	0.0375 ***
			(0.014)	(0.014)
*Human*				0.7217
				(0.487)
*Constant*	0.8942 ***	−4.4281 ***	−4.9353 ***	−4.8562 ***
	(0.022)	(1.328)	(1.344)	(1.345)
*City FE*	Y	Y	Y	Y
*Year FE*	Y	Y	Y	Y
*R-squard*	0.178	0.184	0.187	0.188
*Observations*	2316	2316	2316	2316

Note: Robust standard errors in parentheses. *** *p* < 0.01.

**Table 5:** Robustness test of changing the time of policy implementation.

	(1)	(2)	(3)	(4)
		1 Year in Advance	2 Years in Advance	3 Years in Advance
*Treated* × *Time*	−0.0935 ***	−0.0621	−0.0555	−0.0357
	(0.030)	(0.040)	(0.039)	(0.032)
*Constant*	−4.8562 ***	−5.3516 **	−5.3438 **	−5.2850 **
	(1.345)	(2.685)	(2.683)	(2.674)
*Control variables*	Y	Y	Y	Y
*City FE*	Y	Y	Y	Y
*Year FE*	Y	Y	Y	Y
*R-squared*	0.188	0.191	0.197	0.196
*Observations*	2316	2316	2316	2316

Note: Robust standard errors in parentheses. ** *p* < 0.05, and *** *p* < 0.01.

**Table 6:** Robustness test of changing the period of the policy implementation.

	(1)	(2)	(3)
	(2010–2016)	(2011–2015)	(2012–2014)
*Treated* × *Time*	−0.0361 ***	−0.0188 **	−0.0153 **
	(0.013)	(0.007)	(0.007)
*Constant*	−2.1298	1.8993	0.9776
	(2.018)	(2.095)	(5.298)
*Control variables*	Y	Y	Y
*City FE*	Y	Y	Y
*Year FE*	Y	Y	Y
*R-squared*	0.093	0.013	0.021
*Observations*	1263	903	542

Note: Robust standard errors in parentheses. ** *p* < 0.05, and *** *p* < 0.01.

**Table 7:** Robustness test with the second batch pilots.

	(1)	(2)	(3)	(4)
*Treated* × *Time*	−0.0773 **	−0.0759 **	−0.0866 ***	−0.0863 ***
	(0.033)	(0.033)	(0.033)	(0.033)
*Industr*		−0.0039	−0.0143	−0.0142
		(0.012)	(0.013)	(0.013)
*Infrastr*		−0.0005	−0.0009	−0.0015
		(0.002)	(0.002)	(0.002)
*Finance*			0.0555 ***	0.0558 ***
			(0.015)	(0.015)
*Lnrgdp*			0.5940 ***	0.5858 ***
			(0.132)	(0.133)
*Human*				0.6407
				(0.489)
*Constant*	0.8837 ***	0.8945 ***	−5.1216 ***	−5.0575 ***
	(0.022)	(0.034)	(1.333)	(1.333)
*City FE*	Y	Y	Y	Y
*Year FE*	Y	Y	Y	Y
*R-squared*	0.177	0.177	0.188	0.189
*Observations*	2301	2301	2301	2301

Note: Robust standard errors in parentheses. ** *p* < 0.05, and *** *p* < 0.01.

**Table 8:** The impact mechanism of WECP on GTFP.

	Crowding-Out Effect				Cost Effect			
Explained Variable	(1)	(2)	(3)	(4)	(5)	(6)	(7)	(8)
	Govetec	Govetec	GTFP	GTFP	Comcost	Comcost	GTFP	GTFP
*Treated* × *Time*	−0.0042 ***	−0.0038 ***	−0.0738 **	−0.0880 ***	0.3825 ***	0.4345 ***	−0.0606 **	−0.0774 *
	(0.001)	(0.001)	(0.030)	(0.030)	(0.144)	(0.147)	(0.024)	(0.047)
*Govetec*			0.0842 **	0.5571 **				
			(0.036)	(0.223)				
*Comcost*							−0.0170 *	−0.0191 *
							(0.010)	(0.012)
*Constant*	0.0030 ***	−0.3571 ***	0.8945 ***	−4.9657 ***	15.4024 ***	−10.0325	1.1442 ***	−4.8324
	(0.001)	(0.046)	(0.022)	(1.362)	(0.091)	(11.341)	(0.181)	(3.701)
*Control variables*	N	Y	N	Y	N	Y	N	Y
*City FE*	Y	Y	Y	Y	Y	Y	Y	Y
*Year FE*	Y	Y	Y	Y	Y	Y	Y	Y
*R-squared*	0.207	0.233	0.172	0.182	0.073	0.080	0.195	0.206
*Observations*	2309	2309	2309	2309	939	939	939	939

Note: Robust standard errors in parentheses. * *p* < 0.1, ** *p* < 0.05, and *** *p* < 0.01.

## Data Availability

The data presented in this study are available on request from the corresponding author.

## Appendix A. The Results of Heterogeneity Analysis

**Table A1:** City size heterogeneity.

	(1)	(2)	(3)	(4)
	Small Cities	Medium-Sized Cities	Big Cities	Mega and Super Cities
*Treated* × *Time*	−0.1594 ***	−0.0604 **	−0.0526	−0.0259
	(0.202)	(0.028)	(0.044)	(0.197)
*Lnrgdp*	0.4598	0.8639 ***	0.1821	0.4954
	(0.587)	(0.240)	(0.141)	(1.037)
*Human*	−3.7644	1.5364 *	0.4404	−4.4396
	(2.769)	(0.915)	(0.481)	(4.102)
*Finance*	0.0607	0.1197 ***	0.0154	0.0395
	(0.080)	(0.030)	(0.012)	(0.119)
*Industr*	−0.0375	0.0262	0.0641 ***	0.1487
	(0.027)	(0.028)	(0.019)	(0.696)
*Infrastr*	−0.0170 ***	0.0000	0.0006	0.0393
	(0.005)	(0.004)	(0.002)	(0.035)
*Constant*	−3.5082	−8.0380 ***	−0.9921	4.5047
	(5.913)	(2.389)	(1.434)	(10.703)
*City FE*	Y	Y	Y	Y
*Year FE*	Y	Y	Y	Y
*R-squared*	0.147	0.108	0.122	0.201
*Observations*	313	827	1087	89

Note: Robust standard errors in parentheses. * *p* < 0.1, ** *p* < 0.05, and *** *p* < 0.01.

**Table A2:** Regional heterogeneity in cities.

	(1)	(2)	(3)	(4)	(5)
	East	Central	West	RB	Non-RB
*Treated* × *Time*	−0.0139	−0.1270 *	−0.2096 **	−0.1087 **	−0.0982
	(0.043)	(0.075)	(0.092)	(0.053)	(0.061)
*Human*	0.4680	0.9401	0.5135	0.8358	−0.7318
	(1.081)	(1.481)	(1.377)	(0.846)	(2.930)
*Lnrgdp*	0.3192	0.5705	0.9154 *	0.5443 *	0.4925
	(0.217)	(0.487)	(0.555)	(0.319)	(0.436)
*Infrastr*	−0.0005	−0.0068	0.0023	0.0007	−0.0038
	(0.002)	(0.011)	(0.008)	(0.005)	(0.003)
*Industr*	0.0226	0.0678 *	−0.0241	−0.0876	0.0272
	(0.027)	(0.039)	(0.025)	(0.057)	(0.023)
*Finance*	0.0840	−0.0027	−0.0845 *	0.0510	−0.0157
	(0.062)	(0.038)	−0.05	(0.043)	(0.090)
*Constant*	−2.4719	−4.9908	−8.3224	−4.6853	−4.1366
	(2.276)	(4.978)	(5.488)	(3.264)	(4.407)
*City FE*	Y	Y	Y	Y	Y
*Year FE*	Y	Y	Y	Y	Y
*R-squared*	0.219	0.132	0.290	0.185	0.216
*Observations*	1292	579	445	1421	895

Note: Robust standard errors in parentheses. * *p* < 0.1, ** *p* < 0.05.
